# Impact of a mobile APP-based education model on pediatric food allergy outpatient infusion therapy

**DOI:** 10.1097/MD.0000000000044713

**Published:** 2025-10-31

**Authors:** Hui Hong, Huimin Lang, Jie Zhang, Zixiang Liu

**Affiliations:** a Hangzhou Children’s Hospital Infusion Room, Hangzhou, Zhejiang, China; b Department of Internal Medicine, Hangzhou Children’s Hospital, Hangzhou, Zhejiang, China; c Department of Pediatric Orthopedic Surgery, Hangzhou Children’s Hospital, Hangzhou, Zhejiang, China.

**Keywords:** complications, food hypersensitivity, health education, mobile health applications, outpatient care

## Abstract

This study evaluates the efficacy of an allergy management mobile application (APP) in enhancing outpatient infusion therapy for children with food allergies. Clinical data were retrospectively collected from the electronic medical records of pediatric patients who received outpatient infusion therapy between January and December 2023. Participants were categorized into 2 groups: the APP group (utilizing a hospital-developed allergy management APP) and the non-APP group. Outcome measures included daily crying frequency, duration of crying episodes, time to symptom relief, incidence of complications (mild and severe), and scores on the Disease Awareness and Caregiving Competency Scale for family members. Among the 126 enrolled cases (63 per group), the APP group showed better results than the non-APP group. Specifically, the APP group exhibited fewer daily crying episodes (*P* < .05), shorter crying duration (*P* < .05), faster symptom improvement (*P* < .05), and a lower overall complication rate (*P* < .05). Additionally, caregivers in the APP group achieved higher disease awareness scores and better performance in emotional regulation, role adaptation, lifestyle adjustment, and caregiving skills (all *P* < .05). Implementing an APP-based education model in outpatient infusion therapy for children with food allergies can accelerate symptom resolution, reduce complications and distress behaviors, and improve caregiver knowledge and competence. This approach offers a promising strategy to optimize clinical and psychosocial outcomes in this population.

## 1. Introduction

Food allergy, a common and high-incidence allergic disease during childhood, refers to adverse health effects caused by specific immune reactions that occur repeatedly after exposure to certain foods.^[1]^ Studies have shown that the incidence of food allergies in children aged 0 to 2 years ranges from 5.6% to 7.3%, and the prevalence has been increasing in recent years, drawing significant attention from society.^[2]^ This condition can be classified into 3 types: IgE-mediated, non-IgE-mediated, and mixed-type food allergies.^[3]^ Common food allergens in children include milk, peanuts, eggs, nuts, soy, and shellfish.^[4]^ Clinically, the manifestations are nonspecific and can affect various systems such as the skin, mucous membranes, respiratory tract, digestive tract, and cardiovascular system, leading to symptoms related to the affected organs.^[5]^ The severity of the reactions can range from mild, such as skin itching, to severe life-threatening conditions like anaphylactic shock.^[6]^

Currently, the treatment options for food allergies mainly include avoidance of allergic foods, medications, and intravenous therapy. However, due to the lack of understanding of the disease and treatment by some children and their families, poor adherence during the treatment process often results in suboptimal therapeutic outcomes, which directly affect the recovery of the child.^[7]^ Health education involves the dissemination of health knowledge, the provision of health information, and educational activities to promote individual and community health awareness and behavior. Mobile terminal applications (APPs), which support mobile phones, monitoring devices, and other wireless communication tools, have developed rapidly in recent years and can alleviate the shortage of medical resources, fully meeting the needs of patients for quick diagnosis and self-health management.^[8]^ Studies have shown that APP-based health education models can improve children’s adherence, knowledge retention, and enhance communication between caregivers and patients.^[9]^ However, there is limited research on the effectiveness of APP-based education and management models in children with food allergies.

The purpose of this study is to analyze the effects of using an APP-based education and management model in outpatient intravenous treatment for children with food allergies. Specifically, we aim to retrospectively analyze the impact of this APP-based model on the children’s crying behavior, clinical symptom improvement, complications, parents’ knowledge and caregiving abilities. This research is expected to provide a more effective and scientific educational management plan for children with food allergies, promote their recovery, and offer guidance for clinical practice.

## 2. Materials and methods

### 2.1. General information

Data of outpatient intravenous treatment for children from January 2023 to December 2023 were extracted from the electronic medical record system. The children were divided into 2 groups based on whether they voluntarily used the hospital-developed allergy management APP (recorded in the medical records): the APP group and the non-APP group. Inclusion criteria: diagnosis of food allergy according to the criteria in the “Chinese Pediatric Food Allergy Evidence-based Guidelines,”^[10]^ including the presence of allergic symptoms, a history of allergy or family history of allergy, and positive results from skin tests or serum-specific antibody tests; age between 3 and 7 years; recurrent allergic symptoms for at least 4 weeks; undergoing intravenous therapy; each child had one nurse with a title of nurse or higher responsible for care, and one family member with at least primary school education responsible for caregiving; complete clinical data. Exclusion criteria: severe diseases of organs such as the heart, liver, or kidneys; diseases of the immune, hematological, or other systems; mental illness or intellectual disabilities; severe infections such as viral or parasitic infections; surgery or major trauma history within the 3 months prior to admission. Based on previous studies,^[11]^ it was hypothesized that the use of the APP could reduce the frequency of crying by 20% (α = 0.05, β = 0.2). The required sample size was calculated to be 58 cases per group. A total of 126 cases (63 in each group) were ultimately included, with statistical power of 82%. The study was approved by the hospital’s medical ethics committee, and informed consent was obtained from the children’s families.

### 2.2. Methods

The treatment methods for both groups of children are as follows: 10% calcium gluconate injection (Sichuan Meidakanghua Pharmaceutical Co., Ltd., Meishan, Sichuan Province, China; 10 mL:1 g, National Medicine Standard H51023153) 10 mL and vitamin C injection (Shanghai Modern Harson [Shangqiu] Pharmaceutical Co., Ltd., Shangqiu, Henan Province, China; 2 mL:0.5 g, National Medicine Standard H41024222) 2.0 g are added to 150 mL of 5% saline solution and administered by slow intravenous drip. Additionally, 20 to 60 mL of compound glycyrrhizin injection (Xi’an Yuanda DeYe Co., Ltd., Xi'an, Shaanxi Province, China; 20 mL, National Medicine Standard H20066563) is added to 250 mL of 5% saline solution and administered by slow intravenous drip. During the intravenous treatment, all complications are managed according to standardized protocols, including immediately discontinuing the suspected drug and administering 0.1 to 0.3 mg/kg dexamethasone (Chengdu Tiantai Pharmaceutical Co., Ltd., Chengdu, Sichuan Province, China; 5 mg:1 mL, National Medicine Standard H51020723) intravenously. In cases of severe shock, 0.01 mg/kg epinephrine (Suicheng Pharmaceutical Co., Ltd., Suizhou, Hubei Province, China; 1 mL:1 mg, National Medicine Standard H41021054) is injected.

The non-APP group receives conventional education: first, disease-related education: knowledge about the causes, symptoms, and risks of food allergies is provided to parents through verbal instruction. Parents are advised to avoid exposure to allergens to prevent allergic reactions. Second, dietary education: parents are guided to manage the child’s daily diet, emphasizing the intake of light, easily digestible foods, avoiding irritant foods, and supplementing with nutrients such as vitamin C and vitamin E. Third, intravenous therapy education: before treatment, the purpose, method, precautions, and safety measures of intravenous therapy are explained to the child and their family. During the treatment, parents are required to supervise the child, and any adverse reactions must be promptly reported to the physician. Fourth, discharge guidance: parents are instructed on how to guide the child’s diet and physical activities at home to improve immune function after discharge. In the event of recurrent allergic symptoms, parents are advised to return to the hospital for reevaluation.

The hospital developed the “Pediatric Allergy Management APP” in 2022, which includes a knowledge base on allergies, medication reminders, symptom logs, and other functions. When the child visits for the first time, the nursing staff verbally asks the family if they wish to use the APP, and the usage is recorded in the electronic medical records. The APP group receives education through the APP-based management model: (1) preparation for activities: ① formation of the nursing team: a nursing team is established in the department, led by the head nurse, with 3 responsible nurses as members. The team undergoes training conducted by the head nurse, and after passing an assessment, the team members are allowed to proceed with their work. ② Design of the APP platform: medical and nursing staff in the department develop an APP for managing food allergy children undergoing outpatient intravenous treatment. The APP is used as a mobile education software for this study. Parents and caregivers are guided to scan the QR code to follow the APP and complete the child’s basic information. The platform is available for free use by the families. ③ Data collection and upload: the head nurse arranges for team members to collect images and prepare educational materials. Nurses gather real case images of food allergy children from clinical cases, the internet, and medical books, covering topics such as causes, intravenous therapy, symptoms, complications, exercise, and diet. These materials are uploaded to the APP, enabling responsible nurses to use the APP to better conduct education and management, thereby enhancing the clarity and liveliness of the educational content. (2) Establishing good communication: ① enhancing communication: the team members are required to explain how to use the APP in a warm and friendly manner, quickly establishing a good relationship with the child and family, increasing their trust in the nurse, and offering encouragement and praise to the child. ② Guiding participation: during education through the APP, children and their families are actively encouraged to participate. They are encouraged to ask questions, and the nurse provides advice and solutions based on the child’s specific situation, offering targeted health guidance. (3) APP-based health education content: during different stages of outpatient intravenous treatment, the APP sends educational materials to the child and family. For example, before intravenous therapy, materials on the disease’s causes, therapy, symptoms, and complications are provided. After the treatment, materials on exercise and diet are shared. These materials include both images and written guidance, which parents and children are asked to read and understand. If there are any questions, the nurse will answer and guide them promptly. The content provided through the APP must be easy to understand, colorful, and engaging, with images such as cartoon characters to attract the children’s interest. For children who exhibit crying or resistance during treatment, the images in the APP are used to divert their attention and soothe their negative emotions.

### 2.3. Observation indicators

The following indicators were collected for both groups of children during intravenous treatment: frequency of crying, duration of each crying episode, time to symptom improvement, incidence of mild and severe complications, as well as the family’s disease knowledge and caregiving ability scores: first, *crying indicators*: the hospital’s monitoring system was used to retrieve video recordings of the infusion room. Two independent observers, blinded to the group assignment, recorded the daily frequency and duration of crying episodes for each child. The Kappa consistency coefficient was 0.85. Second, *time to symptom improvement*: This mainly includes the time to improvement for clinical symptoms such as nausea, vomiting, abdominal pain, diarrhea, and skin itching in both groups. Symptom improvement was defined as a reduction in the skin itching score (SCORAD index) by ≥50%, and a reduction in the frequency of gastrointestinal symptoms (vomiting, diarrhea) to ≤1 episode/day, confirmed by a physician.^[12]^ Third, *incidence of mild and severe complications*: this includes the total incidence of mild complications (acute urticaria, allergic rhinitis) and severe complications (asthma, anaphylactic shock). The total incidence of complications is calculated as the number of complications/total number of cases × 100%. Fourth, *family disease knowledge*: a self-developed food allergy knowledge questionnaire was used to assess the knowledge of the family in both groups. The questionnaire covered the disease’s etiology, clinical symptoms, intravenous treatment, prevention of complications, and daily life management. Each item was scored from 0 to 20, with a total score of 100 points. A score ≥ 90 indicated full knowledge, 60 to 80 indicated partial knowledge, and <60 indicated no knowledge. The knowledge awareness rate was calculated as (number of full and partial knowledge/total number) × 100%. The Cronbach α coefficient of the scale was 0.85, and the content validity index was 0.90, indicating good reliability and validity. Fifth, *caregiving ability*: the caregiving ability of the family members was assessed using a pre- and post-intervention caregiving ability evaluation form.^[13]^ The evaluation involved 5 aspects: handling personal emotions, adapting to the caregiving role, adjusting life to meet caregiving needs, coping with changes, and utilizing family and community resources. Each aspect contained 5 items, totaling 25 items. Each item was scored from 0 to 2, with a total score of 50 points. A higher score indicated poorer caregiving ability. The Cronbach α coefficient of the scale was 0.89, and the content validity index was 0.92, indicating good reliability and validity.

### 2.4. Statistical methods

Data were analyzed using SPSS 25.0 statistical software (IBM Corporation, Armonk). Categorical data are expressed as (n [%]), and the chi-square test was used for comparison. Normally distributed continuous data are expressed as ($\bar{x}\pm s$), and the *t* test was used for comparison. A *P*-value of <.05 was considered statistically significant.

## 3. Results

### 3.1. Comparison of baseline data between the 2 groups

There were no statistically significant differences between the 2 groups in terms of gender, age, disease duration, personal allergy history, family allergy history, food allergens, food allergy classification, primary caregivers, or the educational level of the primary caregivers (*P* > .05). See Table 1 for details.

**Table 1:** Comparison of baseline data between the 2 groups.

Indicator		APP group (n = 63)	Non-APP group (n = 63)	*χ^2^*/*t*	*P*
Gender	Male	39 (61.90)	40 (63.49)	0.034	.854
	Female	24 (38.10)	23 (36.51)		
Age (yr)		5.45 ± 1.22	5.58 ± 1.24	0.593	.554
Disease duration (wk)		5.56 ± 0.78	5.62 ± 0.80	0.426	.671
Personal allergy history	Yes	56 (88.89)	54 (85.71)	0.286	.593
	No	7 (11.11)	9 (14.29)		
Family allergy history	Yes	7 (11.11)	9 (14.29)	0.286	.593
	No	56 (88.89)	54 (85.71)		
Food allergen	Milk	27 (42.86)	29 (46.03)	2.125	.547
	Egg/nuts	23 (36.51)	17 (26.98)		
	Wheat	12 (19.05)	14 (22.22)		
	Other	1 (1.59)	3 (4.76)		
Food allergy classification	IgE-mediated	32 (50.79)	36 (57.14)	0.514	.773
	Non-IgE-mediated	14 (22.22)	12 (19.05)		
	Mixed-mediated	17 (26.98)	15 (23.81)		
Primary caregiver	Parent	60 (95.24)	59 (93.65)	0.151	.697
	Other	3 (4.76)	4 (6.35)		
Primary caregiver’s education level	Primary/junior high school	30 (47.62)	28 (44.44)	0.128	.721
	High school or above	33 (52.38)	35 (55.56)		

### 3.2. Comparison of crying indicators between the 2 groups

The APP group had fewer crying episodes per day and shorter duration of each crying episode compared to the non-APP group. There was a statistically significant difference between the groups (*P* < .05). See Table 2 for details:

**Table 2:** Comparison of crying indicators between the 2 groups ($\bar{x}\pm \;s$).

Group	Crying episodes per day (times)	Duration of each crying episode (min)
APP group (n = 63)	1.25 ± 0.34	6.58 ± 1.56
Non-APP group (n = 63)	2.12 ± 0.48	13.30 ± 3.75
* t*	11.740	13.133
* P*	.001	.001

### 3.3. Comparison of symptom improvement time between the 2 groups

The APP group had a shorter time for improvement of clinical symptoms such as nausea, vomiting, abdominal pain, diarrhea, and skin itching compared to the non-APP group. There was a statistically significant difference between the groups (*P* < .05). See Table 3 for details:

**Table 3:** Comparison of symptom improvement time between the 2 groups ($\bar{x}\pm \;s$, d).

Group	Nausea improvement time	Vomiting improvement time	Abdominal pain improvement time	Diarrhea improvement time	Skin itching improvement time
APP group (n = 63)	3.36 ± 0.48	4.16 ± 0.64	3.38 ± 0.50	3.30 ± 0.62	4.24 ± 0.74
Non-APP group (n = 63)	4.25 ± 0.56	5.32 ± 0.72	4.05 ± 0.62	4.11 ± 0.78	5.30 ± 0.85
* t*	9.578	9.558	6.677	6.452	7.465
* P*	.001	.001	.001	.001	.001

### 3.4. Comparison of mild and severe complications between the 2 groups

The total incidence of mild and severe complications in the APP group was lower than that in the non-APP group. There was a statistically significant difference between the groups (*P* < .05). See Table 4 for details:

**Table 4:** Comparison of mild and severe complications between the 2 groups (n[%]).

Group	Mild complications		Severe complications		Total incidence
	Acute urticaria	Allergic rhinitis	Asthma	Anaphylactic shock	
APP group (n = 63)	1 (1.59)	1 (1.59)	0 (0.00)	0 (0.00)	2 (3.17)
Non-APP group (n = 63)	3 (4.76)	3 (4.76)	2 (3.17)	1 (1.59)	9 (14.29)
* χ^2^*	1.032	1.032	2.032	1.008	4.881
* P*	.310	.310	.154	.315	.027

### 3.5. Comparison of disease knowledge awareness between the 2 groups of caregivers

The caregivers in the APP group had a higher level of disease knowledge awareness compared to those in the non-APP group, and the difference between the groups was statistically significant (*P* < .05), as shown in Table 5.

**Table 5:** Comparison of disease knowledge awareness between the 2 groups of caregivers (n[%]).

Group	Complete awareness	Partial awareness	No awareness	Knowledge awareness rate
APP group (n = 63)	39 (61.90)	22 (34.92)	2 (3.17)	61 (96.83)
Non-APP group (n = 63)	28 (44.44)	26 (41.27)	9 (14.29)	54 (85.71)
* χ^2^*				4.881
* P*				.027

### 3.6. Comparison of caregiving ability scores between the 2 groups of caregivers

Before the intervention, there were no statistically significant differences in the caregiving ability scores or total scores between the 2 groups of caregivers (*P* > .05). After the intervention, caregivers in the APP group had lower scores in personal emotion management, adapting to caregiving roles, adjusting life to meet caregiving needs, and total caregiving ability compared to caregivers in the non-APP group, and the difference between the groups was statistically significant (*P* < .05), as shown in Table 6.

**Table 6:** Comparison of caregiving ability scores between the 2 groups of caregivers ($\bar{x}\pm \;s$, points).

Project	Time	APP group (n = 63)	Non-APP group (n = 63)	*t*	*P*
Personal emotion management	Pre-intervention	7.30 ± 1.05	7.18 ± 1.14	0.615	.540
	Post-intervention	5.12 ± 0.76	5.94 ± 0.82	5.821	.001
Adapting to caregiving roles	Pre-intervention	6.06 ± 1.04	5.96 ± 0.95	0.563	.574
	Post-intervention	4.30 ± 0.72	5.20 ± 0.75	6.871	.001
Adjusting life to meet caregiving needs	Pre-intervention	7.14 ± 1.10	6.90 ± 1.22	1.160	.248
	Post-intervention	4.61 ± 0.84	5.38 ± 0.86	5.084	.001
Need for adaptation and providing help	Pre-intervention	6.40 ± 0.92	6.34 ± 1.05	0.341	.734
	Post-intervention	4.68 ± 0.82	4.77 ± 0.90	0.587	.558
Evaluating family and community resources	Pre-intervention	6.56 ± 1.02	6.38 ± 1.25	0.886	.378
	Post-intervention	5.59 ± 0.80	5.71 ± 0.86	0.811	.419
Total score	Pre-intervention	33.42 ± 5.48	32.90 ± 5.56	0.529	.598
	Post-intervention	24.22 ± 4.28	26.98 ± 4.65	3.466	.001

## 4. Discussion

In the past 20 to 30 years, the incidence of allergic diseases has been gradually increasing, with food allergies being one of the major social health issues in children’s nutrition.^[14,15]^ Children, as a vulnerable group for food allergies, can experience allergic symptoms within minutes to 2 hours after eating, and in severe cases, anaphylactic shock can pose a life-threatening risk.^[16]^ Infusion therapy is a primary treatment for food allergies, which can improve the allergic symptoms in children and reduce the occurrence of unnecessary complications.^[17]^ However, due to the recurrent nature of the disease and the long treatment duration, children often exhibit poor compliance, which can negatively impact their prognosis.^[12]^ Therefore, it is of great significance to implement effective education and management during the treatment period. Traditional education methods often suffer from issues such as being too simplistic, lacking specificity, and being difficult to understand, leading to suboptimal educational outcomes. Thus, it is essential to explore more effective and rational models of educational management.^[18]^

### 4.1. The APP-based education management model can improve children’s crying behavior

This study found that the APP group had fewer daily crying episodes and shorter crying durations compared to the non-APP group, indicating that the APP-based education management model can improve the crying behavior of children receiving outpatient infusion treatment for food allergies. The reason for this improvement can be attributed to the use of the APP during the educational management process. Content related to disease etiology, symptoms, infusion treatment, and complications was created in a graphical and textual format and uploaded to the APP for the children and their families to view and read. This approach allowed the children and their families to better understand the disease and treatment-related knowledge, thereby strengthening their adherence to medical instructions and boosting their confidence. Additionally, the educational process paid attention to emotional support for the children, incorporating cartoon character images and other engaging elements, which helped capture the children’s attention and allowed them to undergo infusion therapy in a calm and stable state, thus reducing unnecessary crying.

### 4.2. The APP-based education management model can promote symptom improvement and reduce the occurrence of complications

This study found that the APP group had shorter clinical symptom improvement times and fewer mild and severe complications compared to the non-APP group, suggesting that the APP-based education management model can promote symptom recovery and reduce the occurrence of complications. A study by Pan Ting et al^[19]^ also found that using a WeChat education APP combined with supervised home interventions improved patient treatment adherence and self-management abilities, while reducing the incidence of complications. The reason for these improvements is that during the APP-based education process, children and their families have access to graphical and textual materials in the APP that they can consult anytime and anywhere. By cooperating with medical and nursing staff in areas such as infusion treatment, diet, and exercise, the recovery process is accelerated, leading to a shorter symptom relief time and a significant reduction in disease-related complications. However, the complication rate in this study was relatively low (APP group 3.17% vs non-APP group 14.29%), which may be related to the small sample size. Future studies should expand the sample size for further validation.

### 4.3. The APP-based education management model can improve family members’ knowledge of the disease

This study found that the family members in the APP group had higher disease-related knowledge compared to those in the non-APP group, indicating that the APP-based education management model can enhance family members’ understanding of the disease. A study by Xu Jinling et al^[20]^ similarly found that using an APP for health education can improve patients’ knowledge retention and reduce disease recurrence. As a novel education management model, the APP-based education allows healthcare professionals to upload graphical and textual materials to the APP, providing medical knowledge to both children and their parents in an intuitive and easy-to-understand way. This method helps them fully accept the diagnosis and treatment plans, thereby enhancing their understanding and memory of the disease and its treatment, and ultimately improving their cognitive awareness of the disease and treatment.

### 4.4. The APP-based education management model can enhance family caregiving ability

This study found that after intervention, the family members in the APP group had lower scores in certain caregiving abilities and total scores compared to those in the non-APP group, indicating that the APP-based education management model can improve the caregiving ability of family members. Lei Cheng et al^[21]^ also found that using a mobile APP for health education can enhance family caregiving ability and reduce family members’ anxiety. During the implementation of the APP-based education, children and their families can ask questions to healthcare providers and actively provide feedback on the child’s treatment efficacy and symptom changes. Through thorough and effective communication, a good nurse–patient relationship is established, and family members gain key caregiving skills and knowledge, allowing them to manage the child’s daily care more effectively.

We believe that the APP-based education model holds promise not only for food allergies but also for other types of allergic diseases, such as respiratory and skin allergies. With appropriate modifications, the APP could be adapted to meet the specific needs of patients in various departments, including dermatology and immunology. To promote its broader implementation, collaboration with healthcare providers in different specialties will be essential, along with customizing the educational content to address the unique requirements of each condition. Additionally, successful integration of the APP with hospital information systems and the establishment of adequate training programs for healthcare staff will be critical for its widespread adoption.

### 4.5. Limitations of the study

This study has several limitations. First, the sample was drawn from children with food allergies receiving outpatient infusion treatment at our hospital within a specific time frame, which may limit the generalizability of the findings. As a retrospective cohort study, it is subject to potential confounding factors, such as variations in disease severity and nursing staff differences. While the groups were comparable based on baseline data, differences in disease severity or the care provided by different nurses could influence outcomes like crying behavior and complications. Additionally, certain nursing procedures, like venipuncture techniques, may have affected the children’s emotional responses, particularly during the infusion. Future studies should focus on standardizing these procedures to reduce such variability. The study’s short duration also prevented us from assessing long-term outcomes, such as disease recurrence. Multicenter studies with larger sample sizes are needed to validate these findings. Moreover, while the APP-based education model shows promise, ensuring its sustainability and integrating it with effective nurse–patient communication over time will be important for its long-term success. Additionally, the education levels of caregivers were comparable between the 2 groups, which helps reduce the potential influence of this factor on the study’s results.

## 5. Conclusion

In this retrospective cohort study, the implementation of an APP-based educational management model during outpatient infusion treatment had a positive impact on children with food allergies. It facilitated symptom improvement, reduced the occurrence of complications, decreased the frequency and duration of crying, and enhanced the parents’ knowledge of the disease as well as their caregiving abilities.

## Author contributions

**Conceptualization:** Hui Hong, Zixiang Liu.

**Data curation:** Huimin Lang, Zixiang Liu.

**Formal analysis:** Huimin Lang, Zixiang Liu.

**Investigation:** Jie Zhang, Zixiang Liu.

**Methodology:** Jie Zhang, Zixiang Liu.

**Writing – original draft:** Hui Hong, Zixiang Liu.

**Writing – review & editing:** Hui Hong, Zixiang Liu.
